# Cardiac sodium channel palmitoylation regulates channel availability and myocyte excitability with implications for arrhythmia generation

**DOI:** 10.1038/ncomms12035

**Published:** 2016-06-23

**Authors:** Zifan Pei, Yucheng Xiao, Jingwei Meng, Andy Hudmon, Theodore R. Cummins

**Affiliations:** 1 Department of Pharmacology and Toxicology, Indiana University School of Medicine, 635 Barnhill Drive, Indianapolis, Indiana 46202, USA; 2 Department of Biochemistry and Molecular Biology, Indiana University School of Medicine, 635 Barnhill Drive, Indianapolis, Indiana 46202, USA; 3 Stark Neurosciences Research Institute, Indiana University School of Medicine, 635 Barnhill Drive, Indianapolis, Indiana 46202, USA

## Abstract

Cardiac voltage-gated sodium channels (Nav1.5) play an essential role in regulating cardiac electric activity by initiating and propagating action potentials in the heart. Altered Nav1.5 function is associated with multiple cardiac diseases including long-QT3 and Brugada syndrome. Here, we show that Nav1.5 is subject to palmitoylation, a reversible post-translational lipid modification. Palmitoylation increases channel availability and late sodium current activity, leading to enhanced cardiac excitability and prolonged action potential duration. In contrast, blocking palmitoylation increases closed-state channel inactivation and reduces myocyte excitability. We identify four cysteines as possible Nav1.5 palmitoylation substrates. A mutation of one of these is associated with cardiac arrhythmia (C981F), induces a significant enhancement of channel closed-state inactivation and ablates sensitivity to depalmitoylation. Our data indicate that alterations in palmitoylation can substantially control Nav1.5 function and cardiac excitability and this form of post-translational modification is likely an important contributor to acquired and congenital arrhythmias.

## Abstract (web summary)

Cardiac voltage-gated sodium channels (Nav1.5) are crucial regulators of heart electric activity. Here the authors show that palmitoylation, a process of lipid modification of cysteine residues, modulates Nav1.5 function and affects cardiomyocyte excitability, representing a potential target in treating cardiac diseases.

## Graphical abstract

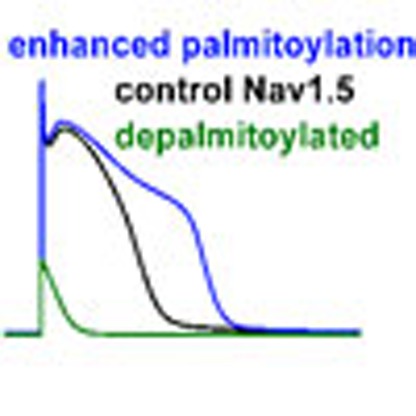

Voltage-gated sodium channels (VGSCs) are responsible for initiation and propagation of action potentials in excitable cells (for example, neurons, muscles and myocytes)^1^,^2^,^3^. VGSCs consist of the α pore forming subunit and one or more β auxiliary subunits that regulate sodium ion flux through the membrane. The cardiac subtype Nav1.5 is predominantly expressed in the heart and plays an essential role in regulating cardiac electric activity^4^. VGSC isoforms can differ in their intrinsic biophysical properties (that is, rapid activation and inactivation), but their activity can also be regulated by post-translational modifications. Although some modifications (such as phosphorylation) have been extensively studied, the regulation of VGSCs by lipid modifications is poorly understood.

Palmitoylation has been recognized as an important post-translational mechanism for the regulation of various membrane proteins, but our knowledge of how palmitoylation regulates ion channels is limited^5^. Protein S-Palmitoylation involves the addition of a 16-carbon palmitic acid chain to an intracellular cysteine residue through a thioester linkage^6^. S-Palmitoylation is a reversible process that can dynamically regulate protein life cycle and function^5^. Multiple enzymes that facilitate the palmitoylation process have been identified in recent years^7^,^8^ and these palmitoyltransferases form a diverse family of proteins (23 members in mammals)^9^,^10^,^11^. The reverse process, depalmitoylation, is mediated by acyl-protein thioesterases^12^. Palmitoylation is thought to play an important role in the biosynthesis pathway of sodium and potassium channels^13^,^14^, trafficking of glutamate channels^15^,^16^ and the spatial organization of aquaporin channels^17^. However, few studies have focused on how palmitoylation directly alters channel biophysical activity at the membrane. Palmitoylation of the intracellular linker between domains II and III in Kv1.1 has been shown to increase the intrinsic voltage sensitivity of the channel^18^. In 1987, brain sodium channel palmitoylation was identified to occur in the early stages of biosynthesis^14^. Electrophysiological data indicates that two putative palmitoylation sites in a Nav1.2 channel clone likely regulate brain sodium channel activity as well as modulating toxin-channel affinity^19^. A general hypothesized mechanism is that the palmitate molecules attached to cysteine residues in ion channels interact with the membrane lipids, changing the lipid membrane environment surrounding the targeted channel as well as impacting protein configuration, thus potentially modulating channel activity.

Although palmitoylation is likely an important regulator of ion channel activity, evidence for the direct influence of palmitoylation on cellular excitability, and its potential involvement in cardiac sodium channelopathies, is lacking. In our study, we show that palmitoylation changes have profound impact on cardiac myocyte excitability. Using both biochemical and electrophysiological assays, we demonstrate that Nav1.5 is palmitoylated in both a heterologous expression system and in native cardiac tissue. We show that modulation by palmitoylation substantially regulates Nav1.5 inactivation, and we identify potential palmitoylation sites regulating this process. A previously identified disease mutation in Nav1.5 associated with cardiac arrhythmia illustrates how the potential loss of palmitoylation at this crucial residue alters channel inactivation and dysregulates myocyte excitability to contribute to cardiac disease. In summary, our experimental data and computer simulation results suggest that palmitoylation not only has profound impact on Nav1.5 activity and cardiac excitability, but is also likely to play a previously unrecognized role in both inherited and acquired cardiac channelopathies. Palmitoylation of Nav1.5 may represent a new target in treating cardiac diseases.

## Results

### Regulation of cardiac excitability by palmitoylation

**Figure 1:**
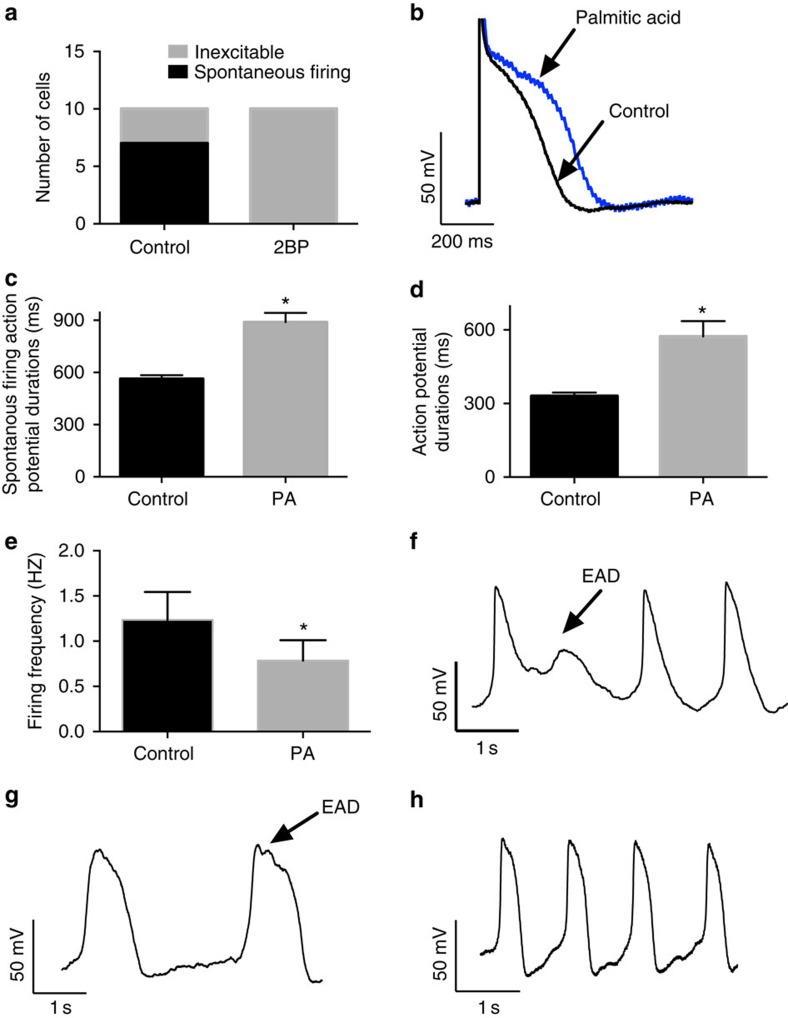
Palmitoylation alters cardiomyocytes excitability. All data are shown as mean±s.e.m. (**a**) Number of cells that are excitable myocytes. Only cells that exhibited inward sodium current were analysed. Seven out of 10 cells (70%) in the control group fired spontaneous action potentials. None of the 10 cells in the 2-Br-palmitate (2BP) group were able to generate action potentials. (**b**) Representative action potential traces of cardiomyocytes with (blue) and without (black) palmitic acid treatment. Palmitic acid(PA) treatment increases the APD. (**c**) Averaged APD measurements from the spontaneous firing myocytes. Averaged APD is 563 ms in the control group and 890 ms in the palmitic acid treatment group. The difference is statistically significant (*n*=5, *P*=0.001 and unpaired Student's *t*-test). (**d**) Averaged APD measurements of single action potential waveform elicited by 2 ms stimulus pulses. APD is 330.4 ms in the non-treatment group and 572.8 ms in the palmitic acid treatment group. The difference is statistically significant (*n*=5, *P*=0.01 and unpaired Student's t-test). (**e**) Firing frequency in control group and palmitic acid treatment group. The firing frequency is 1.2±0.10 in the control group and 0.79±0.11 in the palmitic acid treatment group. The difference is statistically significant (*n*=8, *P*=0.03 and unpaired Student's *t*-test). (**f**–**g**) Spontaneous action potential recorded from palmitic acid treatment group. EAD phenomenon is indicated with arrows. The results are representative of at least three independent experiments. (**h**) Cardiomyocyte spontaneous action potential generated under non-treatment condition. The action potential firing is in a constant rate with no ectopic firing observed.

Cardiac excitability and arrhythmia generation is critically dependent on the gating of multiple ion channel proteins. Recent evidence suggests that the activity of numerous proteins, including ion channels, may be modulated by the palmitoylation, one of the most common post-translational lipid modifications. We asked if manipulating protein palmitoylation in cardiac myocytes would alter excitability. In control myocyte cultures, we routinely observed numerous beating cells on each coverslip and ∼70% of the myocytes that exhibited inward current in voltage-clamp recordings generated spontaneous action potentials in current clamp recordings (Fig. 1a). To decrease protein palmitoylation, we incubated the cells with 2-Br-palmitate (25 μM). 2-Br-palmitate is a nonmetabolizable palmitate analogue that is broadly used as a palmitoyltransferase inhibitor, inhibiting palmitoylation and thus leads to protein depalmitoylation^20^,^21^. Although at least one study demonstrated that 2-Br-Palmitate may also inhibit acyl-protein thioesterase activity^22^, this inhibition is not predicted to be appreciable at 25 μM. After 24 h treatment with 2-Br-palmitate, spontaneously beating myocytes were rarely observed and none of the cells that exhibited inward sodium current in voltage-clamp recordings was able to generate action potentials in current clamp recordings (Fig. 1a). In contrast, we observed distinct effects when myocytes were cultured in the presence of additional palmitate (10 μM palmitic acid), the primary substrate for palmitoylation. Although palmitic acid is structurally similar to 2-Br-palmitate (Supplementary Fig. 1), it does not inhibit palmitoyltransferase. Furthermore, our biochemistry data indicates that excess palmitic acid can enhance Nav1.5 palmitoylation (see below). In contrast to 2-Br-palmitate treatment, palmitic acid did not abolish beating but rather many cells in the palmitic acid treated group displayed spontaneous beating and action potentials that were readily recorded from myocytes in this treatment group. The recorded action potentials from the palmitic acid treatment group exhibited an increased action potential duration (APD) and a decreased firing frequency (Fig. 1b–e) compared with the non-treatment group. Interestingly, when comparing the spontaneous action potential waveforms, we often observed an early after-depolarization (EAD)-like phenomenon in the palmitic acid treatment group (Fig. 1f,g), but not under the control condition (Fig. 1h). These data suggest that reducing palmitoylation in cardiac myocytes attenuates excitability and enhancing palmitoylation (by increasing the availability of substrate) can increase cardiomyocyte excitability.

### Nav1.5 is post-translationally modified by palmitoylation

**Figure 2:**
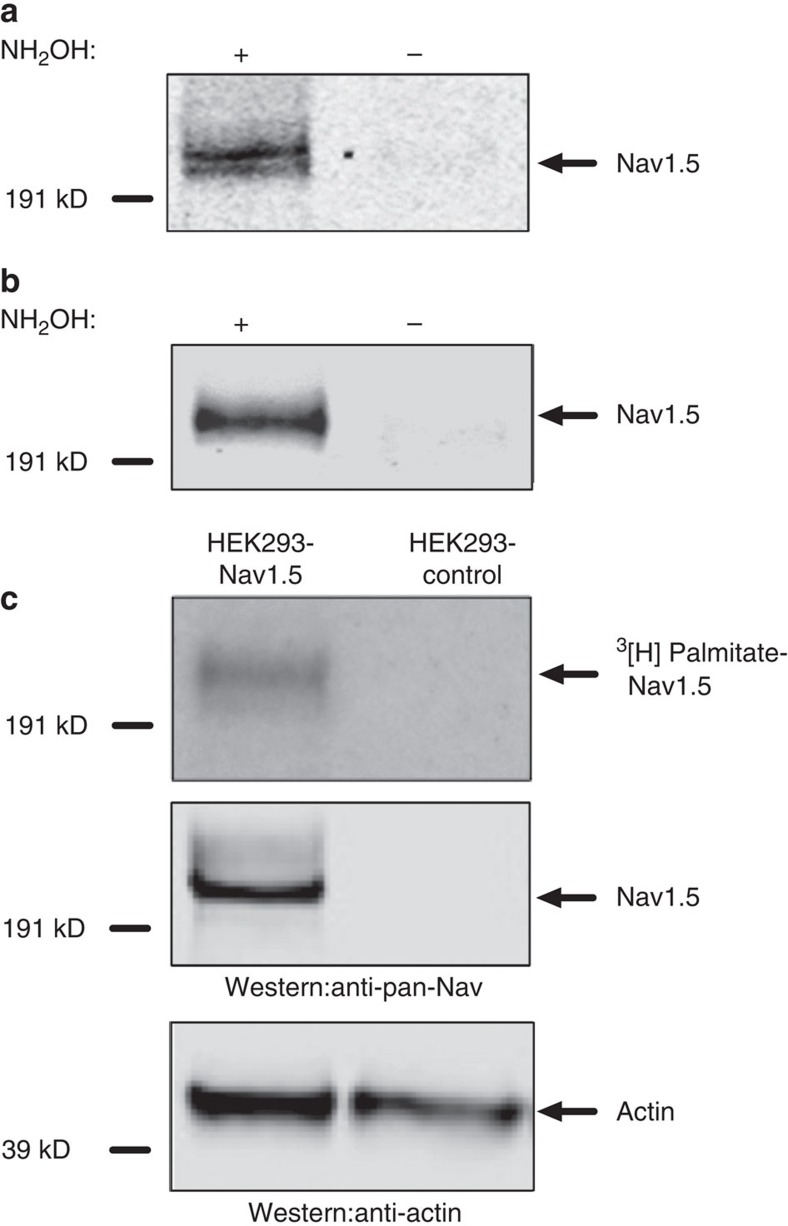
Cardiac sodium channels are post-translationally modified by protein palmitoylation. Three distinct methods were utilized to detect protein palmitoylation: acyl-biotin exchange (**a**,**b**), metabolic tritium labelling (**c**) and click chemistry (Supplementary Fig. 3). The results are representative of at least three independent experiments. (**a**) Nav1.5 is palmitoylated in cardiac tissues, where ‘+' (hydroxylamine treated group) indicates the existence of protein palmitoylation on Nav1.5 channels and ‘−' (tris treated group) serves as the negative experimental control. All the biotinylated proteins (previously palmitoylated proteins) were immunoprecipitated using a streptavidin pull down assay. A sodium channel pan antibody was used to detect transfected or endogenous sodium channel proteins. (**b**) Nav1.5 is also palmitoylated in HEK293 cells with stable expression of Nav1.5 channels. (**c**) Nav1.5 palmitoylation was identified after tritium-labelled palmitic acid treatment. Upper panel: tritium-labelled palmitoylated Nav1.5 was detected in HEK293 cells stably expressing Nav1.5, but not in control HEK293 cells; Middle panel: Nav1.5 was shown in western blot analysis using the same samples; Lower panel: comparable amount of actin from the same samples was shown in western blot analysis.

Our results suggest that enhanced palmitoylation of cardiomyocyte proteins could potentially lead to abnormal cardiac electric activity. Cardiac sodium channels are a critical determinant of electrogenesis in myocytes and therefore we directly investigated if Nav1.5 was palmitoylated using multiple end points. First, we used a non-radioactive acyl biotin exchange (ABE) method^23^,^24^,^25^ that detects thioester-linked protein acyl-modifications, most commonly palmitoylation, in both cardiac tissues and HEK293 cells stably expressing Nav1.5 recombinant channels. Our results indicate that Nav1.5 is S-palmitoylated in cardiac tissues (Fig. 2a, Supplementary Fig. 2a). The signal in the ‘+' (hydroxylamine treatment) lane shows the component captured by streptavidin, which reflects the S-palmitoylated protein before hydroxylamine cleavage. The absence of a signal in the ‘−' (control) lane provides confidence that the signal in the ‘+' lane is indeed dependent on hydroxylamine activity and reflects S-palmitoylation. Caveolin, which has been previously reported to be palmitoylated in cardiac tissue^23^,^26^, was used as a positive control (Supplementary Fig. 2). Calmodulin, which does not contain any cysteine residues, was used as a negative control and shows that there is no signal for non-palmitoylated proteins (Supplementary Fig. 2). Our results also suggest that Nav1.5 is modified by S-palmitoylation in HEK293 cells (Fig. 2b). Although palmitoylation is the most common acylation modification of proteins in mammalian cells, it is not the only possible modification detected by the ABE method. As a direct measure of Nav1.5 palmitoylation, we metabolically labelled HEK293 cells stably expressing Nav1.5 with tritiated palmitate. Nav1.5 was immunoprecipitated and palmitoylation was detected by autoradiography following SDS-polyacrylamide gel electrophoresis. These experiments clearly demonstrated that tritium labelled palmitic acid was incorporated into Nav1.5 channels as identified by immunoprecipitation and western blot analysis (Fig. 2c upper and middle panels). Using a third measurement of palmitoylation, we were also able to detect Nav1.5 palmitoylation with copper-catalysed click chemistry (Supplementary Fig. 3). This method is based on metabolic incorporation of alkynyl derivatives of palmitic acid that can be detected with direct in-gel fluorescence analysis. Altogether, three lines of biochemical evidence support the conclusion that Nav1.5 is indeed palmitoylated in both the heterologous expression system and native cardiac tissue.

### Enhancement and inhibition of Nav1.5 palmitoylation

**Figure 3:**
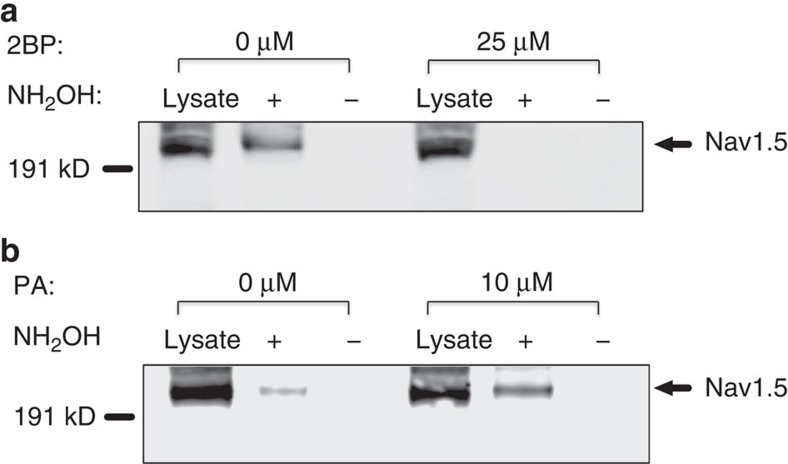
Palmitate lipid treatments alter Nav1.5 palmitoylation in HEK293 cells that stably express Nav1.5. The results are representative of at least three independent experiments. (**a**) 2-Br-palmitate(2BP) treatment inhibited Nav1.5 palmitoylation at 25 μM. (**b**) Palmitic acid(PA) treatment increased Nav1.5 palmitoylation at 10 μM.

To determine if Nav1.5 palmitoylation can be regulated in living cells, we incubated the Nav1.5 stable HEK293 cells with 2-Br-palmitate, the palmitoyltransferase inhibitor that can effectively induce protein depalmitoylation^20^. We treated the Nav1.5 stable cells with various concentrations of 2-Br-palmitate for 24 h and then performed the ABE assay. Results of this experiment indicate that 2-Br-palmitate can eliminate Nav1.5 palmitoylation in HEK293 cells at concentrations as low as 25 μM (Fig. 3a). Next we pre-incubated the cells with palmitic acid for 24 h to increase the substrate of palmitoylation. The results show that the addition of palmitic acid was able to substantially enhance Nav1.5 palmitoylation at concentrations as low as 10 μM (Fig. 3b). Overall, these data demonstrate that the two distinct forms of palmitate exert opposite effects on regulating Nav1.5 protein palmitoylation. These data confirm that Nav1.5 palmitoylation is subject to bidirectional regulation and provided a paradigm that we took advantage of to study how site-specific palmitoylation of Nav1.5 modulates the biophysical properties of the channel.

### Palmitoylation modulates cardiac myocyte sodium currents

**Figure 4:**
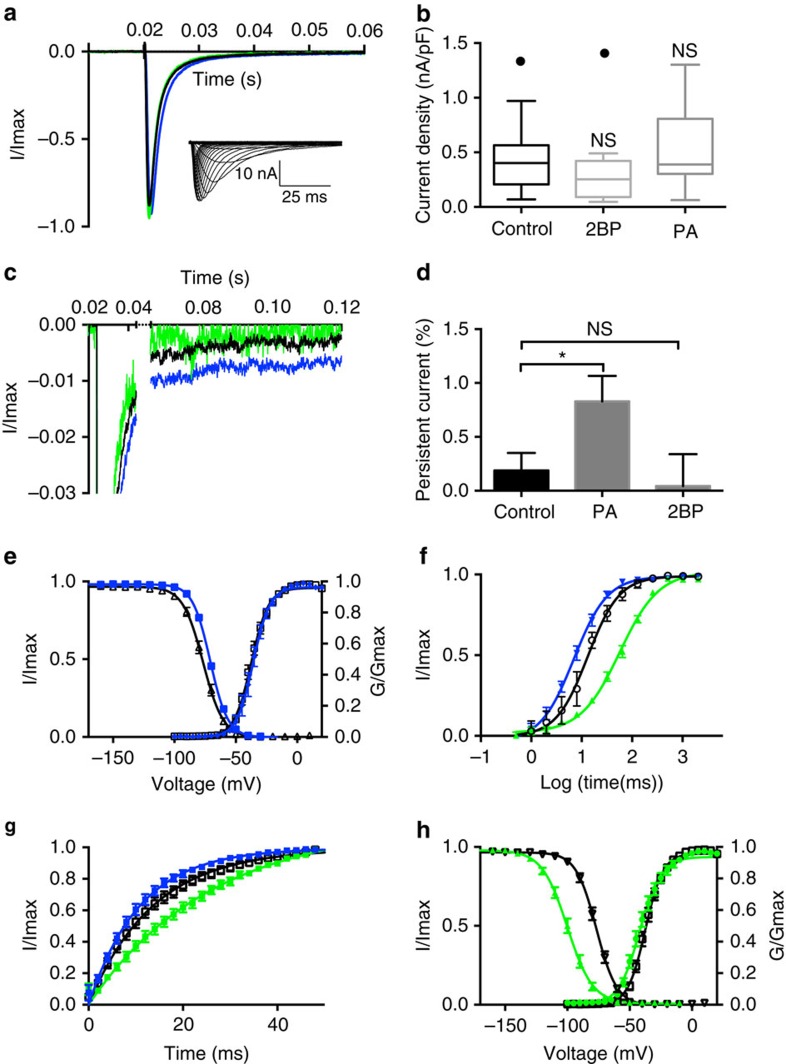
2-Br-palmitate(2BP) and palmitic acid(PA) treatments alter biophysical properties of voltage-gated sodium currents in cardiomyocytes. Data from the 24-h pretreatment of 2-Br-palmitate and palmitic acid are shown in green and blue, respectively. Black lines and open symbols identify data from the control condition. All data are shown as mean±s.e.m. (**a**) Normalized current with and without palmitate lipid treatment. Cells were depolarized to −20 mV from a holding potential of −100 mV. Inset indicates representative traces of Nav1.5 current in cardiomyocytes. (**b**) Comparison of normalized current density. Current density was measured as the peak current divided by cell capacitance (nA/pF). Box and Whisker plots were generated to compare the mean difference. The box was drawn from the 25th to 75th percentiles. Tukey method was used to determine the whiskers and outliers (shown as individual points above the whiskers). In the non-treatment group, mean current density is 0.43±0.04 nA/pF, *n*=35; In the 2-Br-palmitate treated group, mean current density is 0.30±0.08 nA/pF, *n*=16. In the palmitic acid treatment group, the current density is 0.56±0.10 nA/pF, *n*=13. No significant statistical difference is observed in 2-Br-palmitate group (*P*=0.13, unpaired Student's *t*-test) or palmitic acid group (*P*=0.18, unpaired Student's *t*-test). (**c**,**d**) Persistent current (**c**) and statistical analysis (**d**) comparing the control condition and treatment groups. The persistent current was measured as the percentage of peak current. The persistent current is 0.19±0.16% (control); 0.04±0.30% (2-Br-palmitate); 0.83±0.24%(palmitic acid). *n*=6–10. The difference between control group and palmitic acid treatment group is statistically significant (*P*=0.037). (**e**) Comparison of sodium channel voltage dependence of activation and steady-state inactivation between control (black, open symbol, *n*=29) and palmitic acid treatment (blue, *n*=16). (**f**,**g**) Comparison of sodium channel recovery from inactivation with 2-Br-palmitate (*n*=9) and palmitic acid treatment (*n*=9). The recovery time duration is from 0 to 2,048 ms ((**f**) plotted in the log form) and 0–50 ms (**g**). (**h**) Comparison of sodium channel voltage dependence of activation and steady-state inactivation between control (black, open symbol, *n*=29), 2-Br-palmitate treatment (green, *n*=17).

To investigate if bidirectional alterations in the palmitoylation of cardiac sodium channels might contribute to the observed changes in cardiomyocyte excitability, we examined voltage-gated sodium currents in the cultured cardiomyocytes from neonatal rats. In these experiments, we blocked potassium and calcium currents to record isolated voltage-gated sodium currents. Our results demonstrate that neither 2-Br-palmitate nor palmitic acid treatment significantly changed the rate of sodium current fast inactivation (Fig. 4a) or current density (Fig. 4b, *P*>0.05, unpaired Student's *t*-test) in myocytes, suggesting palmitoylation might not play a major role in regulating cardiac sodium channel surface expression. Interestingly, palmitic acid treatment, which enhances palmitoylation of cardiac sodium channels, induced a fourfold increase in the persistent component of the voltage-gated sodium currents (Fig. 4c,d). Although the magnitude of the inward persistent current was slightly <1% of the peak current after palmitic acid treatment, this increase is similar to that observed with several long-QT3 mutations^27^. An increase in these small but powerful late currents is likely to contribute to the prolonged APD- and EAD-like phenomenon shown in Fig. 1. Moreover, palmitic acid treatment shifted the steady-state inactivation to more depolarized voltages (Fig. 4e) and enhanced recovery from inactivation for myocyte sodium currents (Fig. 4f,g), which would also likely contribute to enhanced excitability of cardiomyocytes. On the contrary, 2-Br-palmitate induced a hyperpolarizing shift of steady-state inactivation (Fig. 4h) and slowed the recovery of inactivation for sodium current in cardiomyocytes (Fig. 4f,g). The parameters of activation and inactivation curves, obtained from Boltzmann equation fits, are summarized in Table 1. While palmitic acid treatment induced biophysical changes in sodium current properties that would increase electrical activity in myocytes, the large negative shift in the voltage dependence of inactivation observed with 2-Br-palmitate is predicted to effectively reduce channel availability as well as cardiomyocyte excitability.

### Palmitoylation alters Nav1.5 properties in HEK293 cells

**Figure 5:**
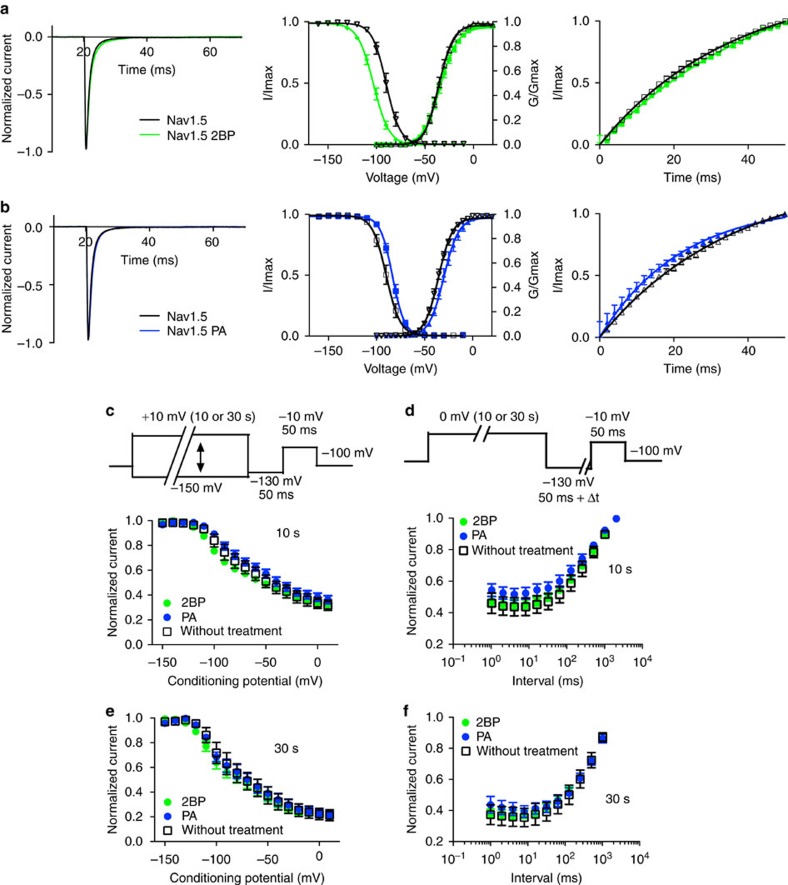
2-Br-palmitate(2BP) and palmitic acid(PA) treatments alter Nav1.5 biophysical properties in HEK293 cells. 2-Br-palmitate treatment group data are shown in green and palmitic acid treatment group data are shown in blue. Control conditions are shown in black lines and open symbols. All data are shown as mean±s.e.m. (**a**,**b**) Left: normalized currents of Nav1.5 compared with 2-Br-palmitate/palmitic acid treatments. Channels were depolarized to −20 mV from a holding potential of −100 mV. Middle: voltage dependence of activation and steady-state inactivation of the channel. Right: Nav1.5 recovery from inactivation. *n*=11. (**c**,**e**) 2-Br-palmitate and palmitic acid treatment does not affect Nav1.5 voltage dependence of slow inactivation ((**c**)10 s; (**e**)30 s; *n*=8). (**d**,**f**) 2-Br-palmitate and palmitic acid treatment does not affect Nav1.5 recovery from slow inactivation ((**d**)10 s; (**f**) 30 s; *n*=8).

To explore the mechanism by which palmitoylation regulates cardiomyocyte sodium currents, we investigated whether modulation of palmitoylation alters the functional properties of Nav1.5 channels expressed in HEK293 cells. The main advantage of these cells is that they do not express significant levels of other ion channels and can be readily used to express recombinant Nav1.5 channels in isolation. We incubated the HEK293 cells expressing Nav1.5 with 25 μM 2-Br-palmitate for 24 h and performed whole-cell electrophysiological recordings. The rate of fast inactivation and the conductance–voltage relationship were not significantly altered by 2-Br-palmitate (Fig. 5a) compared with the control group. However, 2-Br-palmitate treatment substantially shifted the steady-state inactivation of wild-type (wt) Nav1.5 to more hyperpolarized voltages (Fig. 5a, middle). In addition, 2-Br-palmitate treatment slightly slowed the rate for recovery from inactivation (Fig. 5a, right). These effects were also observed with shorter (4 h) 2-Br-palmitate treatments (Supplementary Fig. 4). By contrast, palmitic acid, which enhances Nav1.5 palmitoylation in HEK293 cells (Fig. 3b), shifted the voltage dependence of steady-state inactivation in the depolarizing direction, enhancing channel availability and increased the rate of recovery from inactivation (Fig. 5b). Palmitic acid also slightly shifted the conductance–voltage relationship. Neither 2-Br-palmitate nor palmitic acid seems to affect the Nav1.5 voltage dependence of slow inactivation or recovery from slow inactivation (Fig. 5c–f). The midpoint of the inactivation and activation curves are summarized in Table 1 and representative current traces and current–voltage plots under all three treatment conditions are shown in Supplementary Fig. 5. These results replicate our finding in cardiomyocytes, confirming that Nav1.5 current properties are indeed regulated by modulating palmitoylation and that the HEK293 heterologous expression system serves as a model system to further explore the molecular determinants for this regulation.

### Identification of Nav1.5 cysteines that are palmitoylated

**Figure 6:**
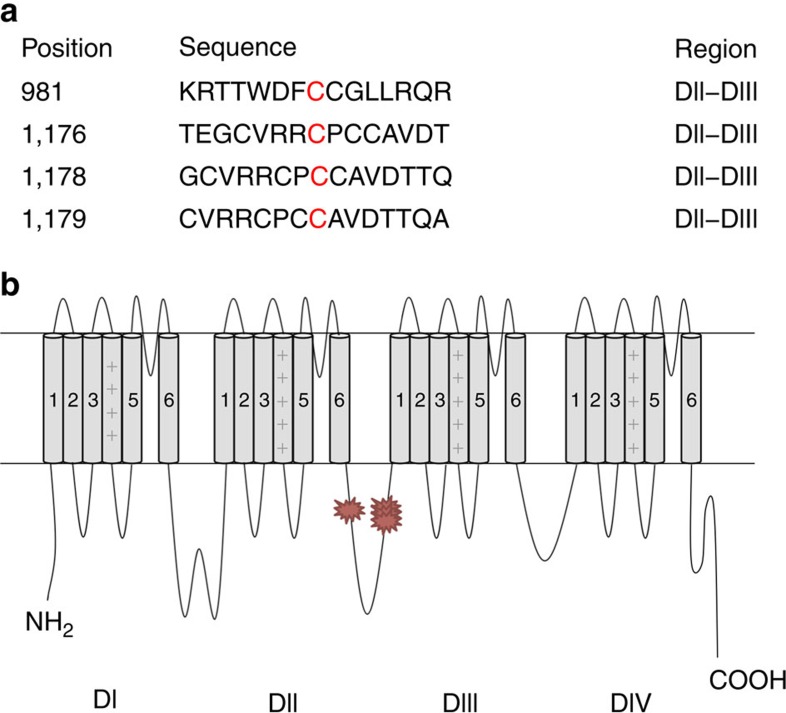
Identification of palmitoylation sites using CSS-palm 3.0. (**a**) Position and sequence of the potential endogenous sites that are predicted by the bioinformatics tool CSS-palm 3.0. (**b**) Schematic view of the localization of four predicted endogenous palmitoylation sites. All four mutations are located on the domains II–III linker area.

To determine how Nav1.5 activity is regulated by palmitoylation, we interrogated the channel sequence for potential modification sites. Although no single conserved sequence of palmitoylation has been identified, the bioinformatics tool CSS-Palm 3.0 can predict which cysteines are potential palmitoylation sites^28^. By comparing the similarity of amino acid sequence surrounding cysteines with experimentally determined palmitoylation sites, the entire Nav1.5 sequence was analysed and four endogenous intracellular cysteine residues that are highly predicted to be palmitoylated were identified (Fig. 6a, Supplementary Table 1). These four cysteines are located in the cytoplasmic linker between domains II and III, and three out of four residues form a cysteine cluster (Fig. 6b). To further investigate the functional role of these four endogenous cysteines in regulating channel palmitoylation, we constructed a mutant channel with these four cysteine residues substituted to alanine residues using site-directed mutagenesis in an attempt to eliminate endogenous palmitoylation. This mutant channel construct, referred to as Nav1.5-AAAA, was used in experiments described below to explore the molecular mechanism contributing to palmitoylation associated functional changes in Nav1.5 activity.

**Figure 7:**
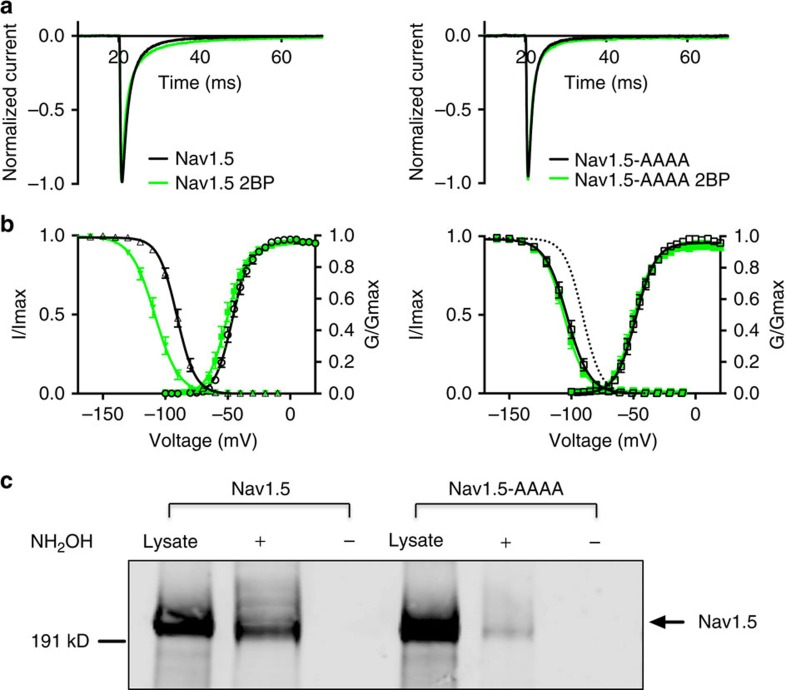
Effects of chemical inhibition of palmitoylation on the biophysical properties of wild-type Nav1.5 and Nav1.5-AAAA. Black lines and open symbols indicate data from control conditions and green indicates data from the 2-Br-palmitate(2BP) treatment group. (**a**) Normalized currents from cells transfected with Nav1.5 (left) and Nav1.5-AAAA (right) with and without 2BP treatment. *n*=7. (**b**) Comparison of wild-type Nav1.5 (left) and Nav1.5-AAAA (right) channel voltage dependence of activation and steady-state inactivation before (black) and after (green) channel depalmitoylation. The dotted line indicates the wild-type Nav1.5 steady-state inactivation. All data are shown as mean±s.e.m. *n*=11. (**c**) Characterization of Nav1.5-AAAA palmitoylation using ABE method. The reduced signal of Nav1.5-AAAA in ‘+' lane (hydroxylamine treated) indicates largely reduced palmitoylation of Nav1.5-AAAA. The results are representative of at least three independent experiments.

To assess the functional role of the four predicted endogenous palmitoylation sites, we transiently transfected the Nav1.5-AAAA channel into HEK293T cells and subsequently treated the cells with 2-Br-palmitate. The results demonstrate that 2-Br-palmitate does not alter Nav1.5-AAAA fast inactivation significantly (Fig. 7a). Wild-type Nav1.5 was transiently transfected into a second set of cells as a control. Treatment with 2-Br-palmitate shifted the steady-state inactivation of transiently transfected wt Nav1.5 in the hyperpolarized direction (Fig. 7b left), similar to that previously observed with the Nav1.5 stable cell line. Interestingly, although chemical inhibition of palmitoylation still shifted steady-state inactivation of Nav1.5-AAAA in the hyperpolarized direction (Fig. 7b, right; Table 1), the effect was substantially smaller (<3 mV) compared with wt Nav1.5 (∼17 mV). The differential effect implies that one or more of these four cysteines are crucial in the regulation of Nav1.5 by palmitoylation. In addition, we found that the midpoint (V½) of Nav1.5-AAAA steady-state inactivation under control conditions was quite close to that of wt Nav1.5 after 2-Br-palmitate treatment, leading us to conclude that the cysteine to alanine substitution(s) alter steady-state inactivation, probably through regulation of palmitoylation on one or more of the four predicted cysteines. To confirm that the ablation of 2-Br-palmitate's effect is due to the alteration in palmitoylation status, we used the ABE assay to assess palmitoylation of wt Nav1.5 and Nav1.5-AAAA channels. Our results indicate that the substitution of cysteine residues to alanine residues largely reduced the palmitoylation of the channel (Fig. 7c). These data strongly support the hypothesis that palmitoylation induced changes in Nav1.5 function is produced by specific modification of one or more of these four endogenous cysteine residues.

### Palmitoylation site associated with Nav1.5 disease mutation

**Figure 8:**
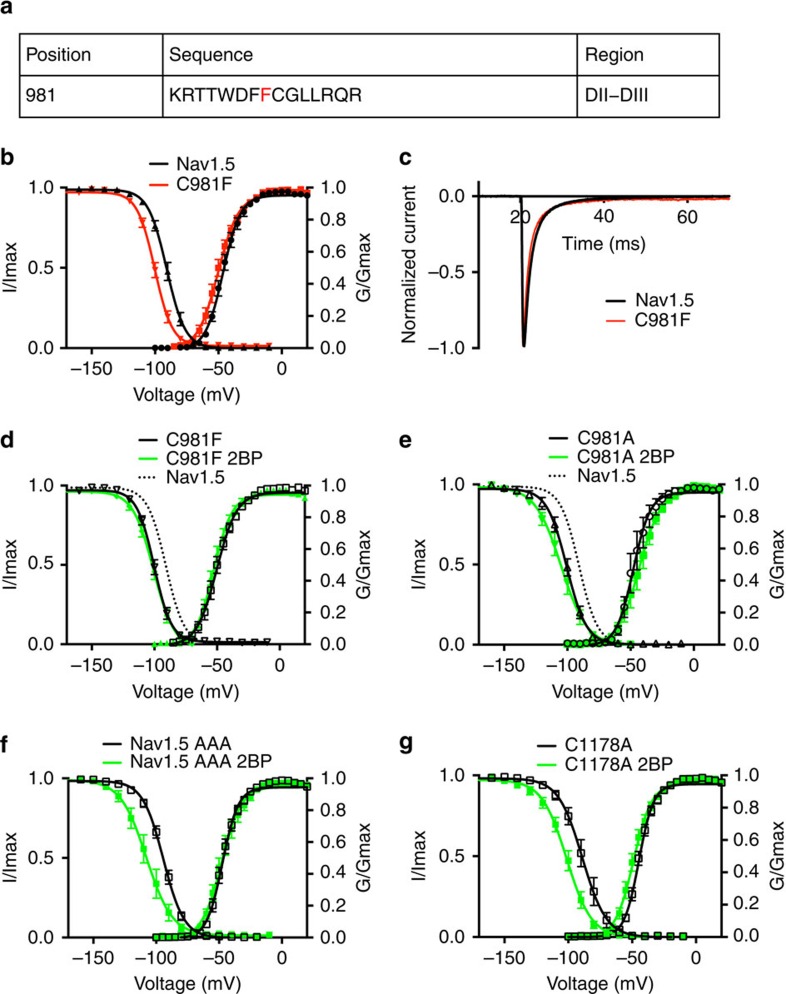
Loss of cysteine mutation from patient with cardiac arrhythmia affects biophysical properties of Nav1.5. All data are shown as mean±s.e.m. (**a**) Long-QT3 mutation (C981F, red) that can potentially abolish the endogenous palmitoylation in wild-type Nav1.5. (**b**) Comparison of voltage dependence of activation and steady-state inactivation of wild-type Nav1.5 (black) and C981F mutant channels (red). *n*=10. (**c**) Normalized current of wild-type Nav1.5 channel (black) and Nav1.5-C981F (red). Current traces were averaged data from 9 measurements. (**d**) Nav1.5-C981F channel activation and steady-state inactivation with (green) and without (black) 2-Br-palmitate(2BP) treatment. *n*=10. (**e**) Nav1.5-C981A voltage dependence of activation and steady-state inactivation with (green) and without (black) 2-Br-palmitate treatment. The dotted line indicates the wild-type Nav1.5 steady-state inactivation. *n*=7. (**f**,**g**) 2-Br-palmitate treatment (green) affect Nav1.5-AAA (**f**) and Nav1.5-C1178A (**g**) voltage dependence of activation and steady-state inactivation. *n*=10.

Numerous missense mutations in Nav1.5 have been identified in individuals with cardiac arrhythmia. In a previously published study, the missense mutation C981F was reported in patient samples submitted for long-QT syndrome genetic testing^29^ (Fig. 8a). Because this mutation occurs at one of the four predicted palmitoylation sites, we developed a Nav1.5 construct containing this mutation (Nav1.5-C981F) and investigated its biophysical properties in HEK293T cells. A significant shift of steady-state inactivation to more hyperpolarized voltages was observed when comparing to wt Nav1.5 (Fig. 8b). No change in the rate of open channel fast inactivation was detected (Fig. 8c) and the C981F mutant channel did not exhibit enhanced persistent current generation. To understand if altered palmitoylation contributes to the shift of the inactivation curve, we treated the Nav1.5-C981F transfected cells with 2-Br-palmitate as in previous experiments. We found that 2-Br-palmitate treatment had very little effect on Nav1.5-C981F channel steady-state inactivation (Fig. 8d). To confirm that the effect is specifically due to the loss of the cysteine residue and not to insertion of a bulky hydrophobic residue, we examined a cysteine to alanine mutation at the same site. Nav1.5-C981A also exhibited a hyperpolarized steady-state inactivation and revealed insensitivity to 2-Br-palmitate treatment (Fig. 8e). Moreover, in our study, both C981F and C981A exhibited very similar properties to the Nav1.5-AAAA channel. This indicates that the C981 site alone accounts for the major palmitoylation effect on Nav1.5 steady-state inactivation, and is likely to be the most important site among the four predicted cysteines. To further confirm the role of the C981 site and investigate the roles of other cysteine residues, we also tested a Nav1.5 mutant with C981 intact and the other three cysteines mutated to alanines (Nav1.5-AAA). Results with this mutant channel indicate that depalmitoylation has a significant effect on Nav1.5-AAA steady-state inactivation (Fig. 8f). This effect was also seen in a single alanine mutation, Nav1.5-C1178A (Fig. 8g). These results further validate the critical and specific role of C981 palmitoylation in regulating channel gating properties.

### Simulations of Nav1.5 palmitoylation and cell excitability

**Figure 9:**
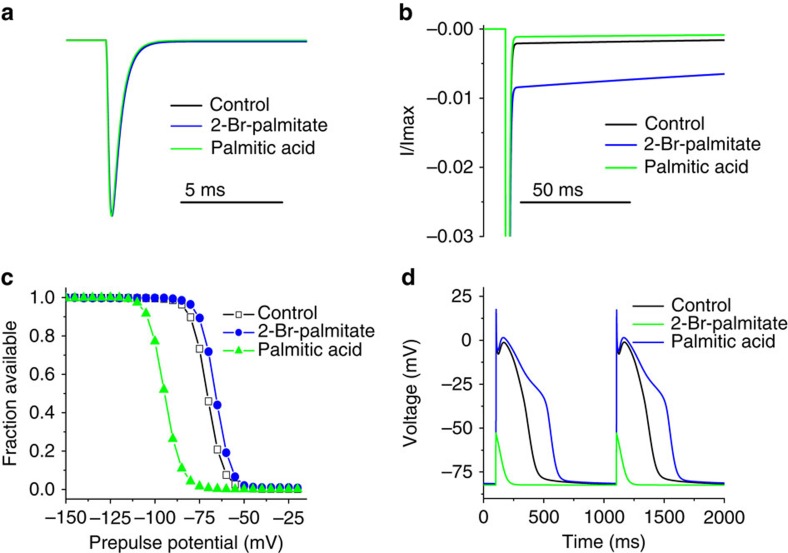
Computer simulations illustrate the impact of depalmitoylation versus enhanced palmitoylation on cardiomyocyte excitability. (**a**) Comparison of peak simulated control Nav1.5, 2-Br-palmitate and palmitic acid ‘treated' currents elicited by a step depolarization to −20 mV. In the simulation, the kinetics of activation and open-channel inactivation were not altered under the three conditions. The current densities were set to be equal to the peak control current density for comparison of kinetic differences. (**b**) Comparison of persistent (or so-called late) Nav1.5 currents for the three simulated conditions. (**c**) Comparison of voltage dependence of steady-state inactivation for control Nav1.5 (black lines and open symbols), 2-Br-palmitate (green lines and symbols) and palmitic acid (blue lines and symbols) ‘treated' currents. (**d**) Simulated action potentials from modelled cardiac myocytes are shown. Action potentials were paced at 1 Hz in these simulations and the second and third action potentials are shown. The black trace shows action potentials with the control Nav1.5 channels, the green trace with Nav1.5 channels modelled to reflect the changes induced by depalmitoylation with 2-Br-palmitate and the blue trace with Nav1.5 channels modelled to reflect the changes induced by enhanced palmitoylation with palmitic acid treatment.

To assess if the observed changes in sodium channel activity might indeed be sufficient to account for the changes in cardiomyocyte excitability shown in Fig. 1, we replicated the 2-Br-palmitate and palmitic acid induced changes in Nav1.5 in a computational model of a human myocyte and assessed the impact on action potential generation. The computational model, previously implemented in the NEURON simulation environment^30^, was minimally adjusted (Supplementary Fig. 6). The consequences of increased palmitoylation (palmitic acid treatment) were simulated by shifting the voltage dependence of inactivation by 5 mV in the positive direction and increasing the non-inactivating component by ∼4-fold (Fig. 9a–c). The consequences of Nav1.5 depalmitoylation were simulated by shifting the voltage dependence of inactivation 24 mV in the negative direction and slightly decreasing the non-inactivating component (Fig. 9a–c). In the model myocyte, the simulated sodium currents with enhanced palmitoylation substantially increased the APD (Fig. 9d). On the contrary, when we simulated the effects of sodium channel depalmitoylation, the model myocyte completely failed to generate action potentials, consistent with our finding in cultured cardiomyocytes (Fig. 1a). This indicates the changes in cardiac sodium channel activity due to alterations in palmitoylation are sufficient to generate the altered excitability that we observed when palmitoylation was manipulated in primary myocyte cultures.

## Discussion

In recent years, the progress of ion channel palmitoylation research has been enhanced by the development of new biochemical and proteomics tools^31^,^32^ and the identification of palmitoyltransferases^10^,^11^. Although a few studies have indicated that VGSCs in the brain are subject to palmitoylation^14^,^19^, it had not previously been determined if sodium channels from other excitable tissues are also subject to modulation by palmitoylation. More importantly, how palmitoylation would affect cellular excitability in any tissue was unclear. This study, for the first time, utilized both biochemical and electrophysiology techniques for investigation of palmitoylation in VGSC modulation. Our data, in conjunction with computer simulations, indicate that palmitoylation can substantially regulate cardiac sodium currents and that alterations in Nav1.5 palmitoylation are likely to play important roles in acquired and inherited cardiac channelopathies.

Palmitoylation is a reversible modification that regulates various steps during protein life cycle, including trafficking, stability and membrane association. Palmitoylation regulation of membrane binding plays a role for many peripheral membrane proteins^33^,^34^ and affects trafficking and surface expression of ion channels^35^,^36^. In our study, palmitoylation induced a small change in current density (Fig.4). However, this difference was not statistically significant, indicating that palmitoylation has negligible impact on cell surface expression of Nav1.5. Instead, our data demonstrate that Nav1.5 palmitoylation has profound impact on channel availability by regulating the voltage dependence of steady-state inactivation in both HEK293 cells (Fig. 5) and cardiomyocytes (Fig. 4). Based on the relative magnitude of the shifts induced by palmitic acid (+6 mV) and 2-Br-palmitate (−20 mV) compared with control experiments, these data could be an indication that as many as two-thirds of the channels are palmitoylated under our control conditions. However, several studies have identified crosstalk between palmitoylation and other post-translational modifications in ion channel proteins^15^,^37^,^38^,^39^ and it is therefore unclear if all Nav1.5 channels are able to be palmitoylated in our experiments.

Four endogenous palmitoylation sites that were likely to regulate Nav1.5 palmitoylation were identified using a palmitoylation prediction algorithm. These four residues are all located in the second major cytoplasmic loop (Fig. 6). Mutation of these four cysteines to alanines had essentially the same effect on steady-state inactivation as 2-Br-palmitate treatment did on wt Nav1.5, and the Nav1.5-AAAA channels were insensitive to modulation by 2-Br-palmitate treatment (Fig. 7), indicating that one or more of these cysteines are responsible for the change in inactivation observed with wt Nav1.5 depalmitoylation. The Nav1.5 C981F and C981A channels had almost the same inactivation properties as the Nav1.5-AAAA channel (Fig. 8), indicating that C981 is likely to be the crucial site at which palmitoylation modulates inactivation of Nav1.5. However, it is unclear how palmitoylation of C981 impacts the voltage dependence of steady-state inactivation. We noticed that all of the sites that are implicated in Nav1.5, as well as the endogenous palmitoylation sites predicted in Nav1.2 to modulate steady-state inactivation, are located in the cytoplasmic linker between domains II and III. One possibility is that by increasing association of this linker to the membrane, palmitoylation may alter the movement of the domain III voltage-sensor, which is important for inactivation^40^. Although our data clearly indicate that C981 palmitoylation is crucial to the gating changes that we observed, we were unable to generate a stable cell line containing only a C981 mutant channel for further biochemical analysis. Therefore we cannot rule out the possibility that one or more of the other three predicted sites are palmitoylated and that this might modulate other properties of Nav1.5 in cardiac myocytes.

Our data also indicate that palmitoylation of Nav1.5 should have a pronounced impact on cardiomyocyte excitability. Inhibiting palmitoylation in myocytes greatly reduced voltage-gated sodium channel availability. In contrast, enhancing palmitoylation increased APD and induced aberrant action potential firing. While multiple cardiac proteins involved in the cardiac action potential might be palmitoylated, our computer simulations indicate that changes in Nav1.5 gating due to palmitoylation are sufficient to cause the excitability changes that we observed in myocytes. However, we still cannot rule out that changes in palmitoylation of potassium and calcium channels might also contribute to the altered action potential activity we observed in myocytes. Despite this potential caveat, the C981F mutation provides additional evidence that alteration of Nav1.5 palmitoylation is likely to impact cardiac action potentials and contribute to arrhythmogenesis. This mutation was identified in the genomic DNA sample of a patient referred for long-QT syndrome clinical genetic testing^29^. In a patient cohort of 2,500 unrelated cases, 110 distinct mutations were identified in *SCN5A* that were absent in the >2,600 control alleles. Although most of the patients were believed to have been referred due to a long-QT phenotype, some were apparently referred because they had Brugada syndrome phenotypes^29^. Based on the biophysical analysis, we predict that the patient with the C981F mutation more likely had a Brugada or overlapping syndrome phenotype, although unfortunately definitive phenotypic data was not available in the study that reported the C981F mutation^29^. Interestingly, we also identified four long-QT3 mutations in the literature (R34C, R1175C, F1473C and R1644C) that our analysis predicts could add an additional palmitoylation site on Nav1.5, suggesting that enhanced palmitoylation may contribute to the biophysical alterations induced by some long-QT mutations. In contrast to C981F effects, Bankston *et al*.^27^ reported that the F1473C Nav1.5 mutation, a long-QT mutation associated with a severe clinical phenotype, had similar biophysical consequences as those we observed with palmitic acid treatment. The F1473C mutation shifted the midpoint of Nav1.5 inactivation in the depolarizing direction by 9 mV and increased persistent (late) currents to ∼0.6% of the peak current. Furthermore, they used a computer model of a ventricular myocyte to show that the combined shift in steady-state inactivation and increase in persistent current amplitude is sufficient to double the duration of the ventricular action potential. All together these data suggest that while depalmitoylation of a key Nav1.5 cysteine can result in reduced cardiac excitability similar to that observed with Brugada syndrome, excessive palmitoylation of Nav1.5 can lead to enhanced cardiac activity similar to that observed with long-QT syndrome mutations.

In summary, our data show that palmitoylation of Nav1.5 can substantially modulate the inactivation properties of cardiac sodium currents and lead to bidirectional alterations in cardiac excitability. The existence of disease-related mutations that alter potential palmitoylation sites indicate that modulation of cardiac sodium channel palmitoylation is likely to be an unrevealed mechanism of cardiac arrhythmia generation. A previous study indicates that palmitoylation of cardiac proteins increases during reoxygenation of anoxic cardiac tissue^41^, and thus enhanced palmitoylation of cardiac sodium channels could contribute to reperfusion induced arrhythmias. Evidence indicates that palmitoylation can also be altered by diet, stress and redox signalling^42^. Although the vast majority of long-QT3 and Brugada syndrome mutations may not directly alter Nav1.5 palmitoylation, increased palmitoylation of Nav1.5 is predicted to enhance EADs and arrhythmias in patients with long-QT3 and reduced palmitoylation of Nav1.5 is expected to exacerbate symptoms in patients with Brugada syndrome; thus regulation of Nav1.5 activity by palmitoylation could be important in multiple cardiac pathophysiological conditions. Brain sodium channel activity is also likely to be regulated by palmitoylation^43^ and altered palmitoylation has been implicated in a number of neurological disorders^44^. Overall, alterations in palmitoylation of VGSCs are likely to have profound effects on cellular excitability in a variety of tissues and pathophysiological conditions.

## Methods

### Chemicals and reagents

2-Br-palmitate and palmitic acid were obtained from Sigma-Aldrich.

### Site-directed mutagenesis

hNav1.5 constructs were introduced into pcDNA3.1(+) with the cytomeglovirus (CMV) promoter using HindIII and XbaI restriction enzyme sites^45^. Specific mutation sites were introduced using the QuikChange II XL site-directed mutagenesis kit according to the manufacturer's instruction. All the constructs were sequenced (ACGT Inc.) to confirm mutation sites.

### Cell culture and transfection

HEK293 and HEK293T cells (ATCC CRL-1573 and CRL-3216, respectively) were cultured in Dulbecco's Modified Eagle Medium (Life Technologies) supplemented with 10% foetal bovine serum (FBS). Cells were transfected using the calcium phosphate precipitation method. Cells were plated on glass coverslips in 24-well plates and the mixture of DNA and calcium phosphate was added to the medium and patch-clamp recordings were performed 24–48 h after transfection.

### Acyl biotin exchange method

Performed as previously described^23^,^25^ with minor modifications. Briefly, cell lysate was treated overnight using *N*-ethylmaleimide to block free cysteines while rotating at 4 °C to ensure complete cysteine alkylation. Next day, the insoluble component was discarded after centrifuge at 13,500*g* for 5 mins. The cell lysate was washed by chloroform–methanol precipitation to remove excess chemicals. The protein interface was washed twice using methanol. After the last centrifugation, protein was dissolved in 500 μl PBS containing 1% SDS. The soluble protein was divided into two equal groups, each containing 250 μl and treated with either NH_2_OH(0.7 M hydroxylamine, 1 mM biotin, 0.2% Triton X-100 and 1 × protease inhibitor) or tris (200 mM tris, 1 mM biotin, 0.2% Triton X-100 and 1 × protease inhibitor) solution. The reaction was carried out in a dark environment for 1 h. The remaining chemicals were removed using a chloroform–methanol wash and resolubilized in PBS containing 1% Triton X-100 and 0.2% SDS. The biotinylated proteins were captured by streptavidin-Sepharose (GE Healthcare) beads in an incubation overnight at 4 °C. The beads were washed four times with PBS containing 1% Triton X-100 and 0.2% SDS the next day. Electrophoresis loading buffer (LDS containing 2% ß-mercaptoethanol) was added to each sample and heated for 10 min at 80 °C.

### Copper (I)-catalysed azide-alkyne cycloaddition reaction (click chemistry)

Untransfected HEK293 cell and HEK293 cells stably expressing Nav1.5 channels were cultured in DMEM with 10% FBS. Cells were then incubated in 17-ODYA labelling media (DMEM with 10% dialyzed FBS (Gemini Bio Products) and 25 mM 17-octadecynoic acid (Cayman Chemical)) for 4 h, washed with D-PBS (Thermofisher Scientific), and lysed with cell lysis buffer (20 mM Hepes, 20 mM NaCl, 5 mM EDTA, 1% Triton X-100 and 1 × protease inhibitor cocktail set V (Millipore)). Cleared lysates were immunoprecipitated with anti-Nav1.5 pan antibody and protein G-Sepharose for 4 h. The beads were washed three times in lysis buffer and resuspended with 44 μl D-PBS. Click chemistry reagents containing1 mM CuSO_4_ (Sigma-Aldrich), 1 mM tris(2-carboxyethyl)phosphine (Sigma-Aldrich), 100 μM tris[(1-benzyl-1H-1,2,3-triazol-4-yl)methyl]amine (Sigma-Aldrich) and 1 μl IRDye 680RD Azide (Thermofisher Scientific) were then added to the beads. After 1 h incubation at room temperature, the beads were washed three times with D-PBS and treated with 1X SDS gel loading buffer. The supernatants were separated into two equal amounts of 25 μl samples. For control experiments, 1.25 μl of 50% stock of hydroxylamine (pH7.0) was added into one of the two equal amount samples to remove the thioester-dependent labelling. The sample mix was then incubated at room temperature for 1 h, heated at 85 °C for 10 min and analysed by standard SDS-polyacrylamide gel electrophoresis gel electrophoresis. In-gel fluorescence of probe-labelled proteins was detected by Li-Cor Odyssey CLx Imager.

### Metabolic labelling and detection of Nav1.5 palmitoylation

HEK293 cells stably expressing human Nav1.5 channels were cultured in 100 mm dishes, while co-cultures of untransfected HEK293 cells were used as negative controls. Each dish was labelled with 120 μCi/ml [^3^H] palmitic acid (PerkinElmer) in regular culture medium overnight. The medium was removed the following day and cells were rinsed three times with PBS. Cell lysates were collected and added to the protein G agarose bead, which was prepared by coupling Nav1.5 pan antibody overnight. After 4 h incubation, the lysate was removed, the protein-G beads were washed three times with lysis butter (20 mM Hepes, 20 mM NaCl, 5 mM EDTA, 1% Triton X-100 and 1 × protease inhibitor cocktail set V(Millipore)), and proteins were eluted from the beads using 4X LDS sample buffer with 2% beta-mercaptoethanol. Samples were then analysed according to the standard denaturing PAGE protocol (Life Technologies, Novex Gel System). The gel was washed three times with hot water and stained with Coomassie Blue for 1 h. To increase the sensitivity of tritium detection, protocols using EN3HANCE (PerkinElmer) was followed based on manufacturer's instructions. To precipitate the fluor in the gel, gel was washed with 10% PEG 800 and 1% glycerol with agitation for 30 min. To further prevent the gel from cracking, the gel was incubated in gel dying buffer (30% Ethanol, 10% glycerol) for 30 min and transferred onto Whatman filter paper for vacuum drying (ThermoSavant). Dried gels were exposed to Biomax high-sensitivity film (Sigma-Aldrich) and stored at −80 °C freezer for ∼3 weeks before developing the film.

### Gel electrophoresis and western immunoblotting

Samples were solubilized in 4 × LDS sample buffer and β-mercaptoethanol. Gel electrophoresis and protein transfer was performed according to the standard protocol (Life Technologies). Sodium channel pan antibody (Sigma, 1:1,000 diluted) was used as a primary antibody to detect Nav1.5 signal. In the cardiac tissue study, caveolin3 antibody (BD Biosciences, 1:5,000 diluted) was used to detect caveolin expression in the cardiac tissues. The fluorescently labelled secondary antibody (IRDye 800CW goat anti-mouse, 1:5,000 diluted) was obtained from Li-Cor. The uncropped scans of gels and western blots of the representative images are shown in Supplementary Figs 7 and 8.

### Whole-cell patch clamp recordings on HEK293 cells

Whole-cell sodium currents were recorded using the method and standard solutions^46^,^47^. Briefly, glass pipettes were pulled and polished with resistance between 0.9 and 2 MΩ.The standard external bathing solution for sodium current recording contained (in mM) 140 NaCl, 1 MgCl2, 1 CaCl2, 3 KCl and 10 HEPES, pH 7.3 adjusted with 1 N NaOH. Osmolarity of the solution was 276 mOsm l^−1^. The standard pipette solution contained (in mM) 140 CsF, 10 NaCl, 1.1 EGTA and 10 HEPES, pH 7.3 (adjusted with CsOH) and the osmolarity of the solution was 293 mOsm l^−1^. Once a gigaohm or greater seal was formed between the cell of interest and the recording pipette, the whole-cell configuration was obtained using gentle suction pulses. Recordings were started ∼5 min after forming whole-cell configuration to allow sufficient equilibration of the intracellular solution and the pipette solution. In the conductance–voltage relationship studies, the cells were held at −100 mV then a 100-ms pulse from −100 mV to +45 mV with 5 mV increments was used to elicit the currents. In steady-state inactivation studies, cells were held at −100 mV and then stepped to an inactivating prepulse from −150 mV to −10 mV with 10 mV increments for 500 ms. The channels that remain available after each inactivating prepulse were evaluated by the peak current produced during a test pulse to 0 mV for 20 ms. In the recovery from inactivation protocol, 500 ms depolarization pulse to +20 mV was induced to fully inactivate the channel, and recovery was assessed by a test pulse from −100 to 0 mV with a varying time between pulses (0–50 ms).

### Cardiac tissue lysate preparation

The use of animals was in compliance with the Guide for the Care and Use of Laboratory Animals published by the US National Institutes of Health and was approved by the Indiana University School of Medicine Animal Care and Use Committee. For ABE experiments, heart tissue (300 mg) from Sprague Dawley rats was homogenized in 10 ml lysis buffer (20 mM Hepes, 20 mM NaCl, 5 mM EDTA, 1% Triton X-100 and 1 × protease inhibitor cocktail set V). The homogenate was centrifuged at 4 °C at 13,200*g*, and the supernatant was collected. Aliquots of supernatant were stored at −80 °C.

### Cardiomyocyte isolation and culture

Experiments were performed following previous protocols^47^, with slight modifications. Sprague Dawley rat pups of postnatal day 1 were used in the harvest, isolation and culture of rat cardiomyocytes. PBS containing 0.5 mg ml^−1^ collagenase was used as the digestion solution. The ventricle tissues were cut into pieces and digested in the 37 °C water bath for 45 min with a gentle mix every 5 min. The cell suspension was collected every 15 min and fresh digestion solution was added each time. Collected cells were plated in 10 cm cell culture dishes for 2 h to allow fibroblast cells attach and then transferred to 24-well plates with gelatin pre-coated coverslips. Cells were incubated under normal cell culture conditions (37 °C, 5% CO_2_) in Dulbecco's Modified Eagle Medium supplemented with 10% FBS. After 24 h incubation, the culture medium was replaced with a low serum medium (0.5% horse serum, 0.5% FBS) to prevent hypertrophy. Cardiomyocytes were cultured for up to 72 h.

### Whole-cell patch clamp recordings on cardiomyocytes

The extracellular solution of whole-cell voltage-clamp recordings contains (mM): 120 NaCl, 1.2 MgCl_2_, 1.5 CaCl_2_, 10 Hepes, 10 tetraethylammonium-chloride, 5.0 sucrose, 5.0 glucose and 0.05 CdCl_2_, pH was adjusted to 7.4 with NaOH. Osmolarity of the solution was 283 mOsm l^−1^. Tetraethylammonium-chloride and CdCl_2_ were used to block potassium and calcium currents in myocytes to focus on recording whole-cell sodium currents. The pipette solution contained: 120 CsF, 5.0 NaCl, 2.0 MgCl_2_, 10 Hepes and 10 EGTA. pH was adjusted to 7.3 with CsOH (osmolarity of the solution was 276 mOsm l^−1^). For the whole-cell current clamp recordings on cardiomyocytes, the bath solution contained (mM): NaCl 126, KCl 5.4, Hepes 10, NaH_2_PO_4_ 0.33, MgCl_2_ 1.0, CaCl_2_ 1.8 and glucose 10. pH was adjusted with NaOH to 7.3 (osmolarity of the solution was 300 mOsm l^−1^). The pipette solution used in current-clamp studies contained (mm): KCl 20, potassium aspartate 110, MgCl_2_ 1.0, Hepes 5.0, EGTA 10 and Na_2_-ATP 5.0. pH was adjusted to 7.2 with KOH (osmolarity of the solution was 295 mOsm l^−1^). Action potentials were elicited by 2 ms stimulus pulses at a frequency of 1 kHz. Spontaneous action potential generation was recorded given the current input at 0 pA. Action potential measurements were started 5 min after establishing the whole-cell configuration.

### Cardiac myocyte simulation

Computer models of cardiac AP firing^48^,^49^ were employed to simulate the impact of the consequences of the changes in channel gating induced by sodium channel palmitoylation. The cardiac myocyte model was adapted from that previously implemented by Ingemar Jacobson in the NEURON simulation environment^30^ (which is available at http://senselab.med.yale.edu/ModelDB/ShowModel.asp?model=3800). The only changes made to the mathematical formulations used in the model were to the voltage-dependent sodium current (*Naf* in the original model). We ran simulations with (1) wild-type Nav1.5 currents, (2) Nav1.5 currents with simulated 2-Br-palmitate treatment and (3) Nav1.5 currents with simulated palmitic acid treatment. A Markov model of Nav1.5 (ref. 50), based on the Hodgkin-Huxley formulation of Nav1.5 in the original cardiac model of AP firing^48^,^49^, was used as it is more amenable to implementation of the palmitic acid and 2-Br-palmitate effects. This formulation also included slow inactivation states. Supplementary Fig. 6 shows a diagram for the Markov model of the simulated Nav1.5 conductance and Supplementary Table 2 shows the transition rate expressions for Nav1.5 and the currents simulating the two treatment groups.

### Data Analysis

Whole-cell patch clamp data were analysed using the Pulsefit (v 8.65, HEKA Electronic), GraphPad Prism (GraphPad Software Inc.) and origin pro 8 (Originlab Corp). All data points are shown as mean±s.e.m., and *n* is presented as the number of the separate experimental cells. Steady-state activation and inactivation curves were fitted using Boltzmann equation: I(V)=Offset+{amplitude/[1+exp((V−V_1/2_)/Z)]}, in which V_1/2_ represents midpoint of activation and inactivation curve. V stands for the test potential and Z represents the slope factor. Two tailed, Student's *t*-tests were used to study the statistical difference of the channel functional parameters with and without channel depalmitoylation, including current density, half maximal activation/inactivation, slope factor, recovery rate. *P* values <0.05 was considered as statistically significant difference.

### Data Availability

The data that support the findings of this study are available from the corresponding author on request.

## Additional information

**How to cite this article:** Pei, Z. *et al*. Cardiac sodium channel palmitoylation regulates channel availability and myocyte excitability with implications for arrhythmia generation. *Nat. Commun.* 7:12035 doi: 10.1038/ncomms12035 (2016).

## Supplementary Material

Supplementary Information Supplementary Figures 1-8 and Supplementary Tables 1-2

## Figures and Tables

**Table 1:** Midpoint voltage of activation and steady-state inactivation with a standard Boltzmann distribution fit

	Activation		Inactivation	
	V1/2 (mV)	K (mV)	V1/2 (mV)	K (mV)
Nav1.5 stable	−33.4±0.3	7.8±0.2	−90.4±0.6	7.1±0.5
Nav1.5 stable 2BP	−34.1±0.4	8.9±0.4*	−103±0.5*	8.1±0.4*
Nav1.5 stable PA	−29.9±0.4	7.7±0.4	−83.4±0.4*	6.3±0.4
Nav1.5 transient	−46.3±0.4	6.9±0.3	−90.8±0.5	7.3±0.4
Nav1.5 transient 2BP	−50.8±0.6	7.8±0.5	−108.4±0.7*	9.7±0.6
Nav1.5-AAAA	−48.2±0.4	8.1±0.4	−103.4±0.7†	9.0±0.6
Nav1.5-AAAA 2BP	−50.4±0.5	7.7±0.4	−106.0±0.5	8.4±0.5
Nav1.5 C981F	−50.2±0.5	8.1±0.4	−100.2±0.5†	6.9±0.4
Nav1.5 C981F 2BP	−52.6±0.4	7.3±0.4	−100.7±0.7	8.3±0.6
Nav1.5 C981A	−47.8±0.7	6.7±0.6	−100.5±0.6†	7.9±0.5
Nav1.5 C981A 2BP	−43.2±0.7	8.5±0.6	−104.8±0.9	10.3±0.8
Nav1.5 cardiomyocytes	−37.3±0.3	7.1±0.3	−76.6±0.5	8.2±0.4
Nav1.5 cardiomyocytes 2BP	−42.2±0.6	8.5±0.5*	−99.4±0.6*	10.1±0.5*
Nav1.5 cardiomyocytes Palmitic acid	−36.1±0.5	6.7±0.5	−71.0±0.5*	7.3±0.5

^*^indicates *P*<0.05 compared with the corresponding non-treatment group. The two-tailed, Student's *t*-test was used to study the statistical significance.

^†^indicates *P*<0.05 compared with wild-type group.

All data are shown as mean±s.e.m.
