# Supplementary material for: Cardiac sodium channel palmitoylation regulates channel availability and myocyte excitability with implications for arrhythmia generation
Source: Nat Commun. 2016 Jun 23;7:12035. doi: 10.1038/ncomms12035 (PMC4931030; doi:10.1038/ncomms12035)
Supplement: Supplementary Information — Supplementary Figures 1-8 and Supplementary Tables 1-2 [file ncomms12035-s1.pdf]

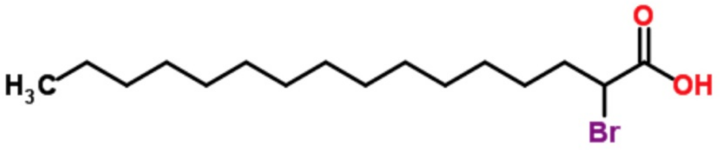

2-Br-palmitate

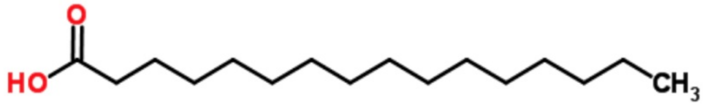

palmitic acid

Supplementary Fig. 1: Structures of 2-Br-palmitate and palmitic acid.

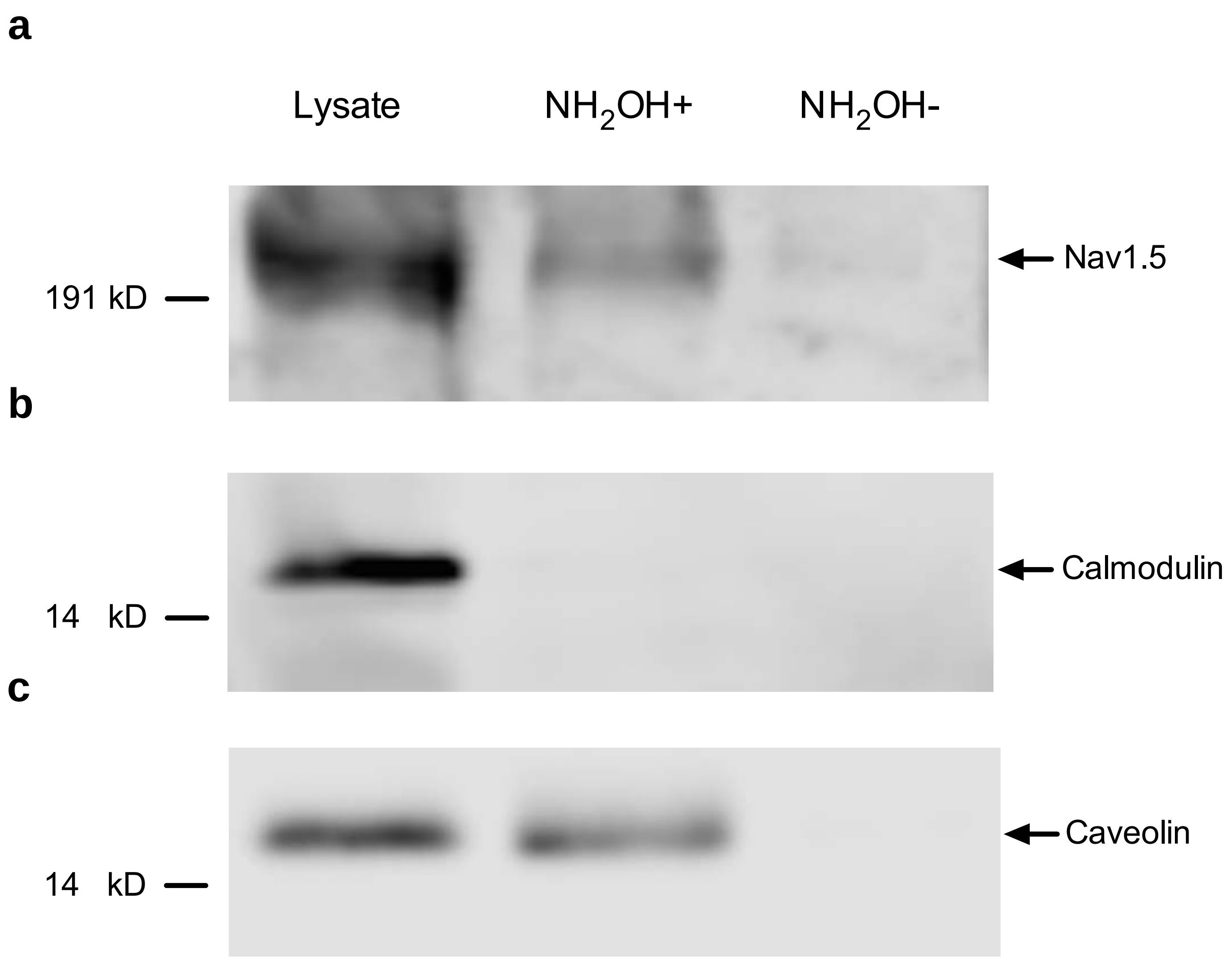

Supplementary Fig. 2: Identification of Nav1.5 palmitoylation using acyl biotin exchange ABE experiment. The results are representative of at least three independent experiments. Left lane indicates total input lysate. The middle lane (NH_2OH+) indicates palmitoylated protein. The right lane (NH_2OH-) indicates the negative control group treated with tris solution. Results suggest Nav1.5 and caveolin is palmitoylated and calmodulin is not palmitoylated.

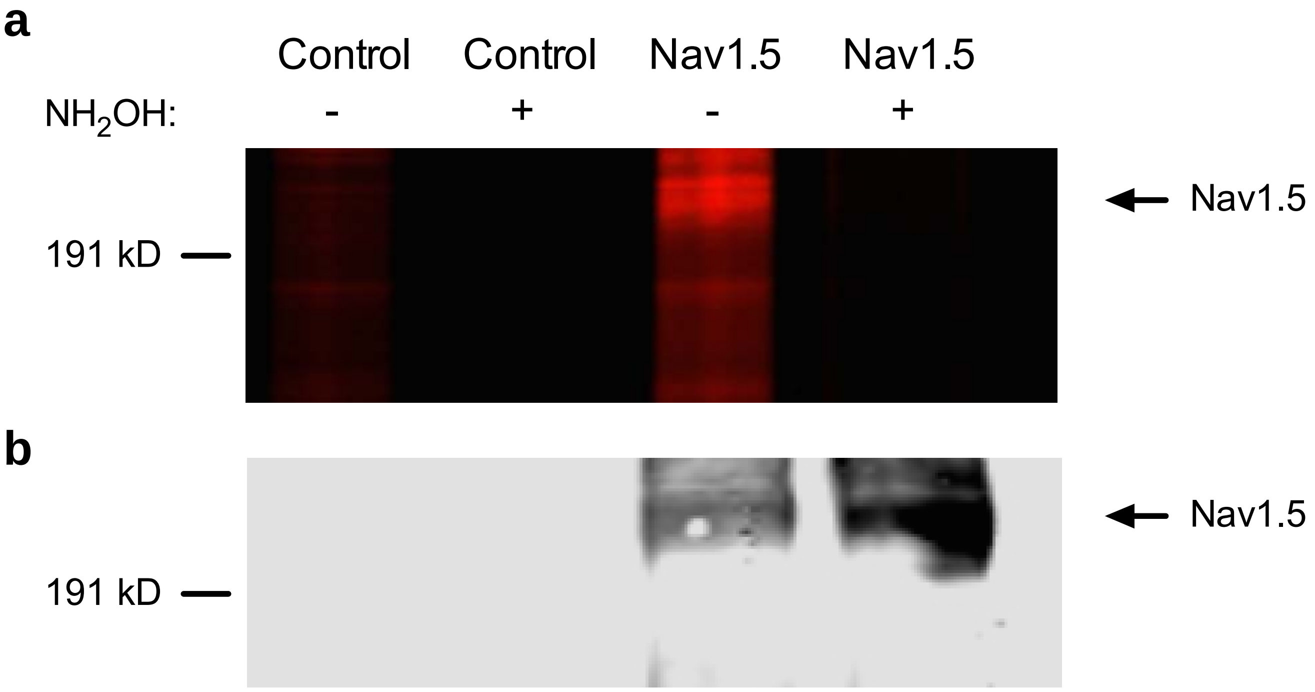

Supplementary Fig. 3: Identification of cardiac sodium channel palmitoylation using copper-catalyzed click chemistry. The results are representative of at least three independent experiments. **(a)** In-gel fluorescence analysis of protein palmitoylation after immunoprecipitation of Nav1.5 from human embryonic 293 (HEK293) cells stably expressing Nav1.5. Red bands indicating palmitoylated proteins visualized by the azide-linked reporter tags after click chemistry reaction. The location of Nav1.5 protein was marked by the arrows. The left two lanes were from untransfected HEK293 cells and the right two lanes were from HEK293 cells with stable expression of Nav1.5. The presence (+) and absence (-) of hydroxylamine was used to demonstrate specificity of thioester-dependent labeling and protein palmitoylation. The absence of palmitoylation signal in '+' group indicates that the azide-activated fluorescent dye which previously attached to the palmitoylated site was removed by hydroxylamine cleavage, confirming the specificity of the palmitoylation signal observed in '-' group. **(b)** Western blot analysis of the samples used in panel (a). Nav1.5 was detected using sodium channel pan antibody in both hydroxylamine treated and untreated samples. Nav1.5 protein was not expressed in untransfected HEK293 samples.

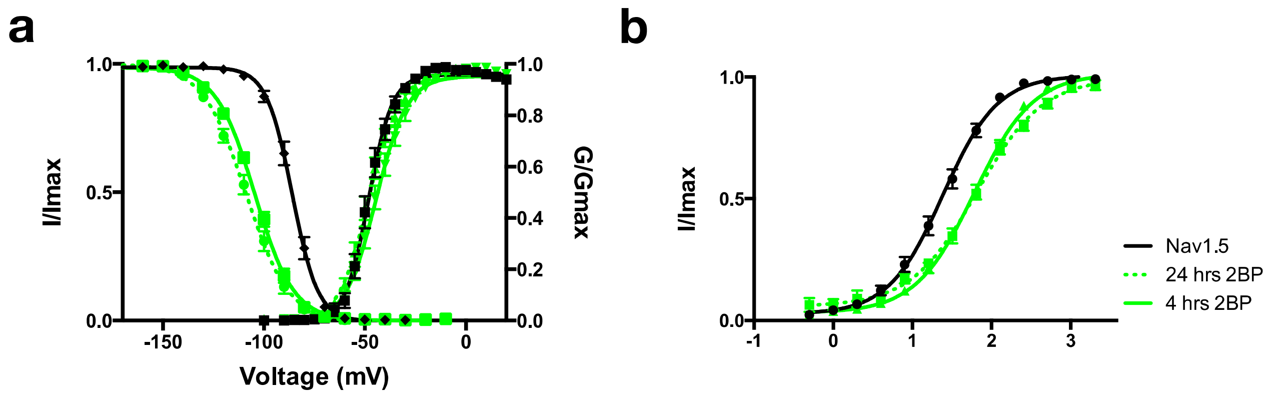

Supplementary Fig. 4: 2-Br-Palmitate effects on Nav1.5 biophysical properties. All data points are shown as mean±s.e.m.. Green lines and symbols indicate 2-Br-Palmitate (2BP) treatment (solid lines show 4 hours treatment and dashed lines show 24 hours treatment) and black lines and symbols indicates no treatment involved. **(a)** Both 24 hours (n=8, p<0.0001, student's t test) and 4 hours (n=8, p<0.0001, student's t test) treatment significantly shift Nav1.5 steady-state inactivation. There is no significant difference between 24 hours and 4 hours treatments (student's t test, p=0.0628). **(b)** Both 24 hours and 4 hours treatment alter recovery from inactivation (n=6).

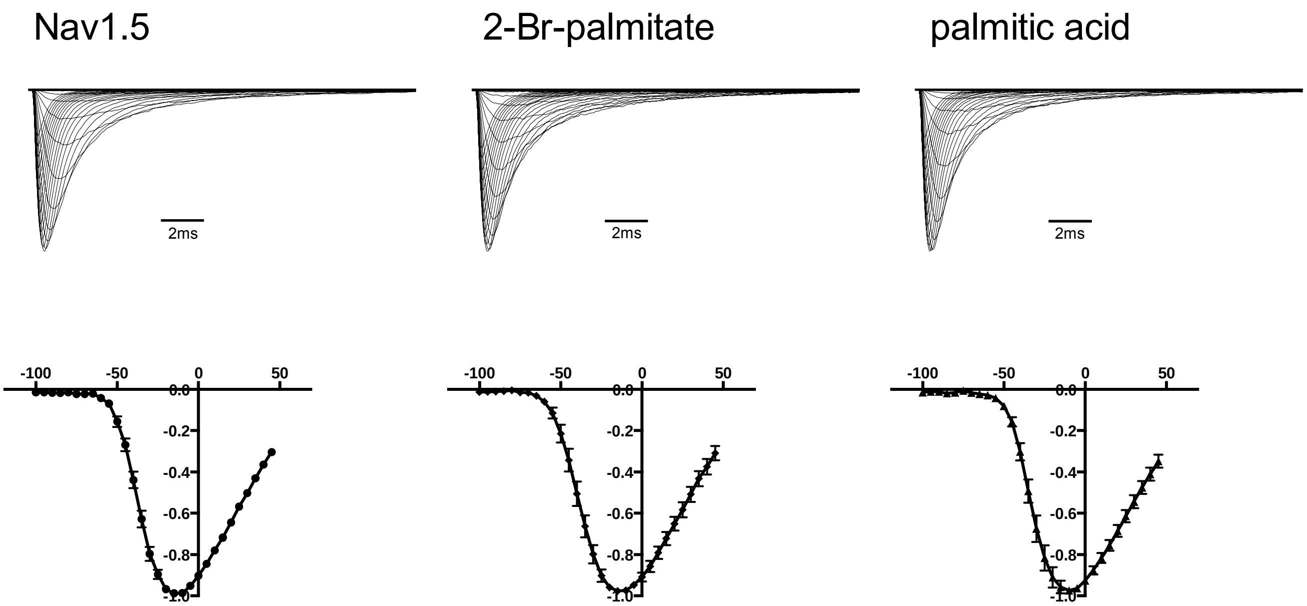

Supplementary Fig. 5: Representative current traces and normalized current-voltage (IV) plot. Left: control; Middle: 2-Br-palmitate treatment; Right: palmitic acid treatment. Top panel shows the representative normalized current traces and the bottom panel shows the corresponding IV curves (n=10, all data points are shown as mean±s.e.m.).

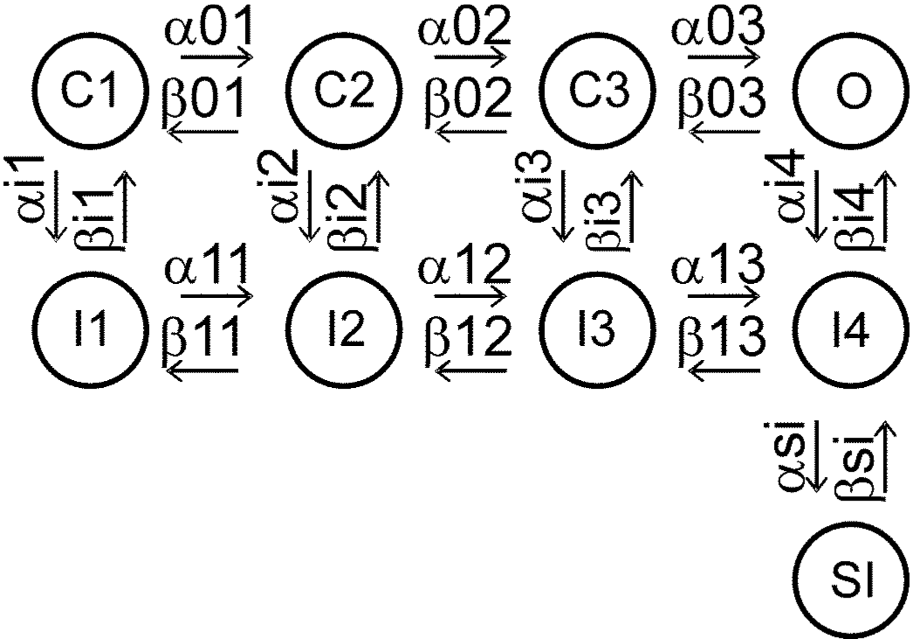

Supplementary Fig. 6: Diagram of Markov model used for computer simulation of voltage-gated sodium channel conductances. C1-C3, closed (non-conducting) states; O, open (conducting) state; I1-I4, fast-inactivated (non-conducting) states; SI, slow-inactivated (non-conducting) state. Forward and reverse transitions are indicated and transition rate expressions are provided in supplemental table S1.

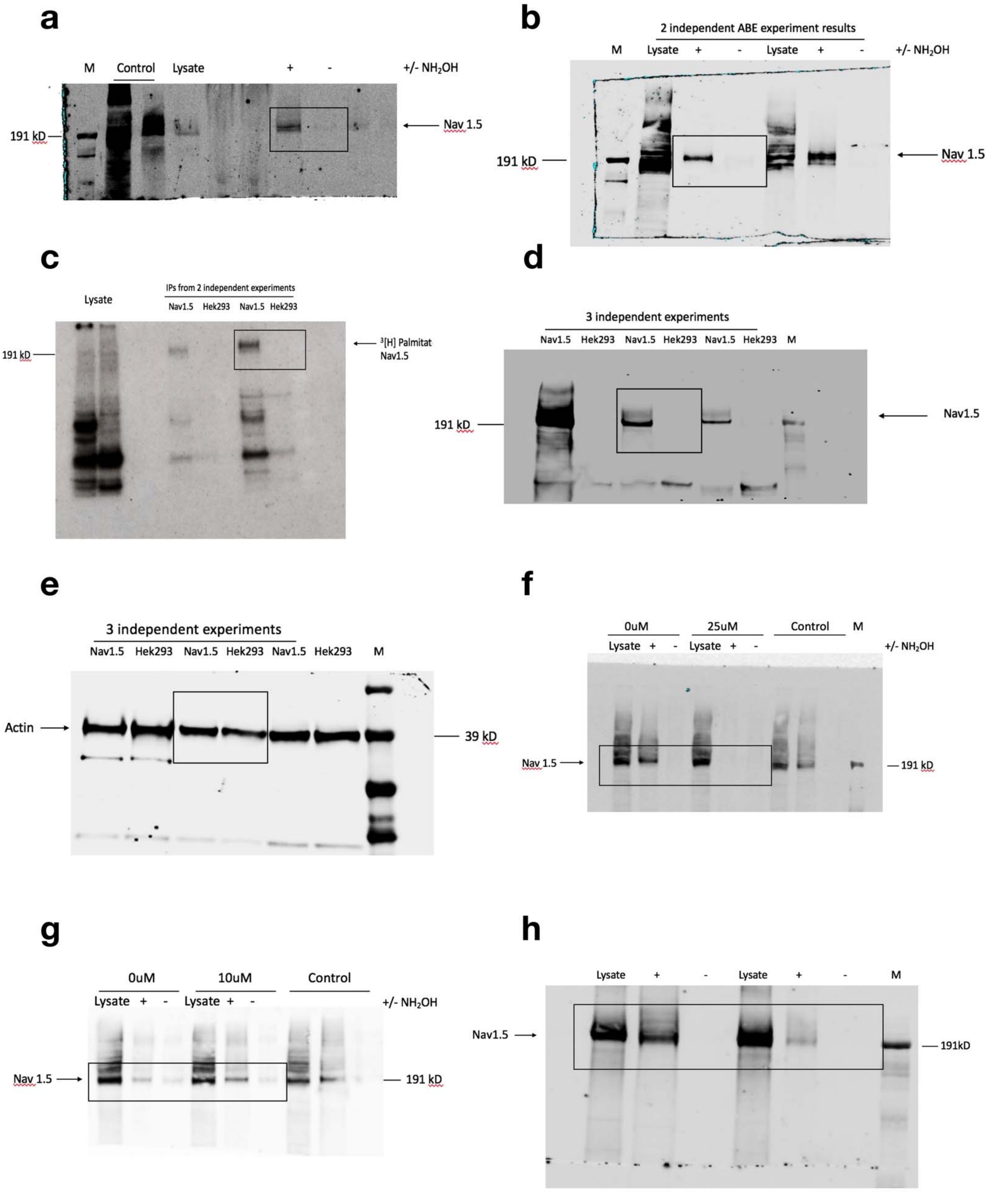

Supplementary Fig. 7: Uncropped films and western blot images from Figure 2 (a-c), Figure 3 (a-b), Figure 7 (c).

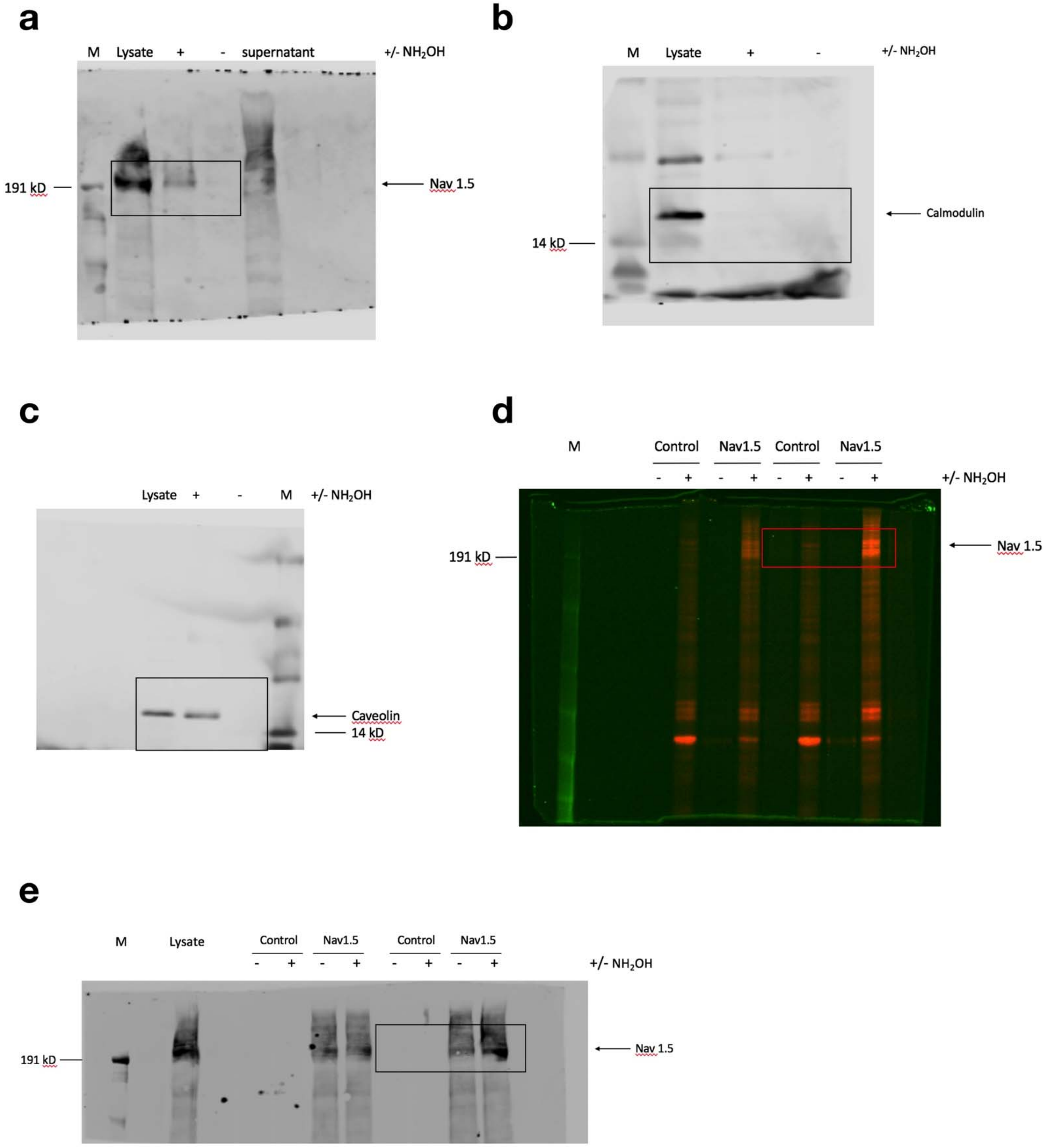

Supplementary Fig. 8: Uncropped gel and western blot images from supplementary Fig. 2 (a-c), Fig. 3 (a-b).

Supplementary Table 1

The score matrix of palmitoylation prediction results from CSS-PALM 3.0. The predicted sites (C981, C1176, C1178, C1179) are indicated with red colored text. The high threshold setting of CSS_PALM was used for the prediction cutoff.

Position	Peptide	Score	Cutoff	Cluster
139	LFNMLIMCTILTNCV	0.286	0.308	Cluster A
145	MCTILTNCVFMAQHD	0.454	0.497	Cluster B
182	KILARGFCLHAFTFL	0.167	0.308	Cluster A
260	VMVLTVFCLSVFALI	0.124	0.308	Cluster A
280	MGNLRHKCVRNFTAL	0.339	1.225	Cluster C
326	GTSDVLLCGNSSDAG	0.281	0.308	Cluster A
335	NSSDAGTCPEGYRCL	0.769	1.225	Cluster C
341	TCPEGYRCLKAGENP	0.364	1.225	Cluster C
373	FRLMTQDCWERLYQQ	0.167	0.497	Cluster B
489	MSSGTEECGEDRLPK	0.167	0.308	Cluster A
597	KKNSTVDCNGVVSLL	0.934	1.225	Cluster C
649	MLTSQAPCVDGFEEP	0.119	0.308	Cluster A
683	LEESRHKCPPCWNRL	0.194	0.497	Cluster B
686	SRHKCPPCWNRLAQR	0.306	0.497	Cluster B
699	QRYLIWECCPLWMSI	0.554	1.225	Cluster C
700	RYLIWECCPLWMSIK	1.116	1.225	Cluster C
726	TDLTITMCIVLNTLF	0.727	1.225	Cluster C
896	LIIFRILCGEWIETM	0.014	0.308	Cluster A
906	WIETMWDCMEVSGQS	0.11	0.308	Cluster A
915	EVSGQSLCLLVFLLV	1.198	1.225	Cluster C
**981**	KRTTWDF**C**CGLLRQR	**1.083**	0.497	Cluster B
982	RTTWDFCCGLLRQRP	1.14	1.225	Cluster C
1004	AQGQLPSCIATPYSP	0.372	1.225	Cluster C
1046	PGDPEPVCVPIAVAE	0.306	1.225	Cluster C
1128	AEPQAPGCGETPEDS	0.289	1.225	Cluster C
1136	GETPEDSCSEGSTAD	0.537	1.225	Cluster C
1167	DVKDPEDCFTEGCVR	0.033	0.308	Cluster A
1172	EDCFTEGCVRRCPCC	0.12	0.497	Cluster B
**1176**	TEGCVRR**C**PCCAVDT	**0.806**	0.497	Cluster B
**1178**	GCVRRCP**C**CAVDTTQ	**0.333**	0.308	Cluster A
**1179**	CVRRCPC**C**AVDTTQA	**0.75**	0.497	Cluster B
1198	WWRLRKTCYHIVEHS	0.306	1.225	Cluster C
1272	KYFTNAWCWLDFLIV	0.496	1.225	Cluster C
1341	IMNVLLVCLIFWLIF	0.11	0.308	Cluster A
1363	FAGKFGRCINQTEGD	0.25	0.497	Cluster B
1384	IVNNKSQCESLNLTG	0	0.308	Cluster A
1539	VTIMFLICLNMVTMM	0.727	1.225	Cluster C
1575	VAIFTGECIVKLAAL	0.694	1.225	Cluster C
1703	TFANSMLCLFQITTS	0.09	0.308	Cluster A
1728	LNTGPPYCDPTLPNS	0.223	1.225	Cluster C
1742	SNGSRGDCGSPAVGI	0.488	1.225	Cluster C
1850	VSGDRIHCMDILFAF	0.471	1.225	Cluster C

Supplementary Table 2

Transition rate expressions for Nav1.5 conductance models.

Transition	For Nav1.5, control conditions	For palmitic acid treated	For 2Br-palmitate treated
α01	-3*0.32*(v+47.13)/(exp(-0.1*(v+47.13))-1)	unchanged	unchanged
β01	0.08 * exp(-(v)/11)	unchanged	unchanged
α02	-2*0.32*(v+47.13)/(exp(-0.1*(v+47.13))-1)	unchanged	unchanged
β02	2 * (0.08 * exp(-(v)/11))	unchanged	unchanged
α03	-0.32*(v+47.13)/(exp(-0.1*(v+47.13))-1)	unchanged	unchanged
β03	3 * (0.08 * exp(-(v)/11))	unchanged	unchanged
α11	-3*0.32*(v+47.13)/(exp(-0.1*(v+47.13))-1)	unchanged	unchanged
β11	0.08 * exp(-(v)/11)	unchanged	unchanged
α12	-2*0.32*(v+47.13)/(exp(-0.1*(v+47.13))-1)	unchanged	unchanged
β12	2 * (0.08 * exp(-(v)/11))	unchanged	unchanged
α13	-0.32*(v+47.13)/(exp(-0.1*(v+47.13))-1)	unchanged	unchanged
β13	3 * (0.08 * exp(-(v)/11))	unchanged	unchanged
αi1	(1/(0.13*(1+(exp(-1*(v+10.66)/11.1)))))	(1/(0.13*(1+(exp(-1*(v-1.66)/11.1)))))	(1/(0.13*(1+(exp(-1*(v+75.66)/11.1)))))
βi1	(0.135*exp(-0.147*(v+80)))	unchanged	unchanged
αi2	(1/(0.13*(1+(exp(-1*(v+10.66)/11.1)))))	(1/(0.13*(1+(exp(-1*(v-1.66)/11.1)))))	(1/(0.13*(1+(exp(-1*(v+75.66)/11.1)))))
βi2	(0.135*exp(-0.147*(v+80)))	unchanged	unchanged
αi3	(1/(0.13*(1+(exp(-1*(v+10.66)/11.1)))))	unchanged	unchanged
βi3	(0.135*exp(-0.147*(v+80)))	unchanged	unchanged
αi4	(1/(0.13*(1+(exp(-1*(v+10.66)/11.1)))))	unchanged	unchanged
βi4	((0.135*exp(-0.147*(v+80)))+0.0022)	((0.135*exp(-0.147*(v+80)))+.01)	((0.135*exp(-0.147*(v+80)))+.0006)
αsi	0.00321	unchanged	unchanged
βsi	(.0037933*0.00001)*exp(-(v)/7.7)	unchanged	unchanged

Transitions are as diagramed in Supplemental Fig. 6. Values are in ms^{-1}.
